# Study on the Fabrication of Super-Hydrophobic Surface on Inconel Alloy via Nanosecond Laser Ablation

**DOI:** 10.3390/ma12020278

**Published:** 2019-01-16

**Authors:** Zhen Yang, Yanling Tian, Yuechao Zhao, Chengjuan Yang

**Affiliations:** 1 Key Laboratory of Mechanism Theory & Equipment Design, Ministry of education, Tianjin University, Tianjin 300350, China; yangzhen1992@tju.edu.cn (Z.Y.); meytian@tju.edu.cn (Y.T.); zhaoyuechao123@tju.edu.cn (Y.Z.); 2 School of Engineering, University of Warwick, Coventry CV4 7AL, UK

**Keywords:** nanosecond laser, wettability conversion, ambient air, airborne hydrocarbons, synergistic effect

## Abstract

Nanosecond laser ablated metallic surfaces showed initial super-hydrophilicity, and then experienced gradual wettability conversion to super-hydrophobicity with the increase of exposing time to ambient air. Due to the presence of hierarchical structures and change of surface chemistry, the laser-induced Inconel alloy surfaces showed a stable apparent contact angle beyond 150° over 30-day air exposure. The wetting states were proposed to elucidate the initial super-hydrophilicity and the final super-hydrophobicity. The basic fundaments behind the wettability conversion was explored by analyzing surface chemistry using X-ray photoelectron spectroscopy (XPS). The results indicated that the origins of super-hydrophobicity were identified as the increase of carbon content and the dominance of C–C(H) functional group. The C–C(H) bond with excellent nonpolarity derived from the chemisorbed airborne hydrocarbons, which resulted in dramatic reduction of surface-free-energy. This study confirmed that the surface chemistry is not the only factor to determine surface super-hydrophobicity. The laser-induced super-hydrophobicity was attributed to the synergistic effect of surface topography and surface chemical compositions. In this work, the corresponding chemical reaction was particularly described to discuss how the airborne hydrocarbons were attached onto the laser ablated surfaces, which reveals the generation mechanism of air-exposed super-hydrophobic surfaces.

## 1. Introduction

As a precipitation-hardenable super-alloy, Inconel 718 alloy (IN718) has attracted more attention due to its significant potentials in gas turbines, rocket motors and spacecraft. This material shows perfect high strength, thermal and wear resistance in serious conditions [1,2]. However, IN718 alloy is regarded as a difficult machining material by conventional techniques due to its low material removal rate, high shear strength, and excessive tool wear [3]. Previous literatures have demonstrated that surface modification with a super-hydrophobic property plays an important role in various applications, for instance, self-cleaning [4,5], anti-bacteria [6], anti-corrosion [7], enhanced heat transfer [8], and drag reduction [9,10]. It is therefore urgent to find alternative method to manufacture IN718 alloy with super-hydrophobic property to expand its value in potential applications.

By investigating materials from natural creatures including lotus leaves and butterfly wings, two main factors for achieving super-hydrophobic surfaces are rough micro/nano structures and the low-free-energy coatings [11]. Up to now, many scholars design functional interface with super-hydrophobicity using the approaches of thermal embossing [12], sol-gel [13], chemical etching [14,15], electrodeposition [16,17], chemical vapor deposition [18], and laser ablation [19,20,21,22,23,24].

Among the above-mentioned methods, ultrafast laser ablation is intensively used to produce micro/nano-scaled devices for various applications including micromechanics [25], optics [26], optomechanics [27], and microfluids [28,29]. Particularly, laser process is considered as a facile technique to directly obtain super-hydrophobic surfaces on varieties of materials [30]. Laser ablation results in stable 3D binary rough structures through precise control, which is necessary to prolong surface durability [31]. For instance, Sun et al. successfully fabricated the super-hydrophobic silicon wafers with various regular surface patterns employing excimer laser, showing an apparent contact angle of 163 ± 1° [32]. Kietzig et al. used femtosecond laser to produce microstructures on several pure metallic surfaces. The ablated substrates initially presented super-hydrophilic property. With the aging time exposed to ambient air, the contact angle reached about 160° with very small hysteresis [33]. In our previous paper, the titanium substrate was ablated by nanosecond laser, the fabricated surface also showed initial super-hydrophilicity. After being exposed to air for approximately 30 days, the laser-induced titanium substrates presented slow wettability conversion to final super-hydrophobic state [34]. It is worthwhile to mention that there are some differences of the time-dependent contact angle hysteresis between the nanosecond laser-induced surfaces and the femtosecond laser-induced surfaces. For example, Rung et al. showed an increased contact angle hysteresis over time after the femtosecond laser ablation [35], while Yang et al. presented a decreased contact angle hysteresis after the nanosecond laser ablation [36]. These findings can enable laser ablation to various applications, such as ink-jet printing, immersion lithography [37], self-cleaning [38], and so on.

Many previous papers reported that surface topography and surface chemistry contributed to the super-hydrophobicity of the laser ablated surfaces [39,40]. However, the relationship between laser-induced surface topography and surface chemistry was seldom investigated. By exploring the fresh and aged pristine IN718 surfaces as well as the laser ablated IN718 surfaces, we investigated the mechanism of time-dependent apparent contact angle and contact angle hysteresis, and found that the surface chemistry was by no means the single factor to determine laser-induced super-hydrophobicity.

In this study, the IN718 substrates were ablated with line or grid pattern by nanosecond laser. The as-prepared surfaces initially presented a super-hydrophilic nature and then reached a steady super-hydrophobic state over 30 days air exposure. Surface morphology was observed by scanning electron microscopy (SEM), indicating that the laser-induced binary rough structures amplified the initial super-hydrophilicity based on the Wenzel theory. The roughness-based explanation was also proposed to elucidate the level of final super-hydrophobicity. The variations of surface chemistry detected using X-ray photoelectron spectroscopy (XPS) implied that the air-exposed wettability conversion could be ascribed to the increase of carbon content and reduction of surface polarity, due to the chemisorbed organic matters from air moisture. Particularly, the fresh and aged flat-IN718 substrates were investigated in this research, which revealed that the laser-induced super-hydrophobicity was attributed to the synergistic effect of surface roughness and chemisorbed organic substances.

## 2. Materials and Methods

### 2.1. Materials

The experiments were performed on the IN718 material (its chemical components were presented in Table 1) with a dimension of Φ 20 mm × 1 mm. The cleaning solutions of ethanol as well as acetone with analytical grade were provided by Beijing Chemical Works (Beijing, China). A commercial water purification system (Hitech Instruments Co., Ltd, Shanghai, China) provides the distilled water. All chemicals without further purification were used in the following experiments. Prior to laser irradiation, the average surface roughness (*R*_a_) of the IN718 substrates was confined below 1 μm by mechanical polishing. After polishing process, many dirty contaminants (e.g., carbon substance) were attached on the polished samples. These contaminants should be removed by ultrasonic cleaning in acetone, ethanol solution and distilled water for 5 min in sequence because they have a significant effect on the laser ablation process. In addition, the spontaneous formation of oxide layer on pristine IN718 substrates was not wanted since it would influence surface oxidation process during laser ablation. Therefore, the IN718 substrates were activated in 10 wt. % HCl for 60 s to remove surface oxide layer. Before the samples were irradiated by nanosecond laser, they were washed by distilled water and quickly dried by nitrogen flow. 

### 2.2. Laser Processing

**Figure 1:**
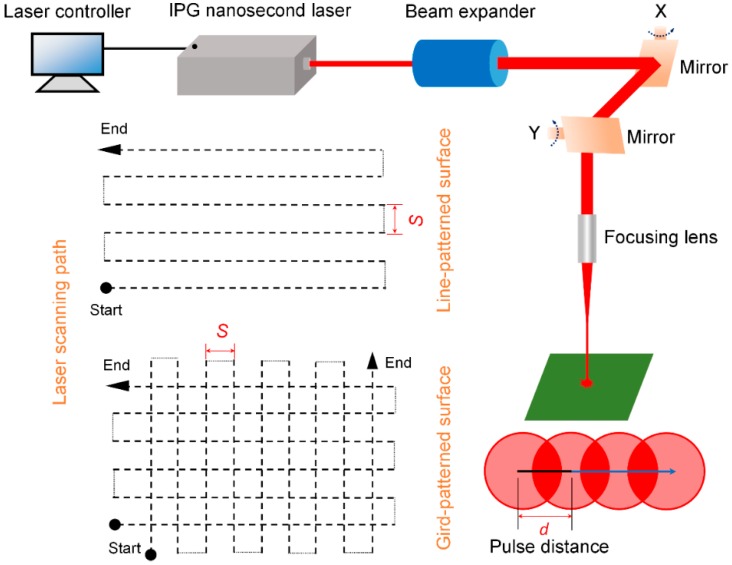
Laser system components and schematic explanation of laser pulse distance.

A nanosecond laser system (IPG photonics, Burbach, Germany) was used to irradiate the prepared IN718 substrates. The components of the laser system are displayed in Figure 1, and the selected laser parameters are presented in Table 2. The focused laser beams were delivered by a focusing lens which was placed in the scanning head. The moving laser beams scanned the substrates, first along the *x* (0°) axis achieving the line-patterned structures, and then along the *y* (90°) axis achieving the gird-patterned structures. 100 μm was set as the spacing (*S*) between two successive laser scanning lines. All the experiments were carried out under ambient air condition. 

### 2.3. Measurement and Characterization

Surface morphology of the laser processed IN718 surfaces was observed using SEM (FEI, Helions G4 CX, Hillsboro, OR, USA). 3D profiles were measured using a laser confocal microscopy (Olympus, LEXT-OLS4000, Tokyo, Japan). As defined by Marmur [41,42], measurements of the apparent contact angle (APCA), the advancing and receding contact angles were carried out using a contact angle meter (AST, VCA optima, Billerica, MA, USA) with a 3 μL distilled water droplet. Here, the APCAs were recorded everyday under normal lab condition. Five different locations were measured to make the data reliable and reproducible. The advancing contact angle (*θ*_a_) and the receding contact angle (*θ*_c_) were measured by increasing and decreasing the volume of water droplet, respectively. When the water droplet volume increases (or decreases), the contact line still keeps pinned until the advancing contact angle (or receding contact angle) is received. Further increase (or decrease) of the volume of water droplet will cause the movement of contact line, whereas the contact angle remains the same [43]. It is defined that the difference between the advancing contact angle and the receding contact angle is contact angle hysteresis (*θ*_a_–*θ*_c_). The fresh pristine IN718 substrate (denoted as IN-i) was also studied for reference. Another pristine IN718 substrate was exposed to air condition beyond one year in cleanroom, which was denoted as IN-ii. Over 30 days air exposure, both the line- and grid-patterned surfaces reached super-hydrophobic state that were abridged as abridged as IN-iii and IN-iv, respectively. XPS (Thermo Fisher Scientific, Escalab 250Xi, Waltham, MA, USA) was employed to detect surface chemistry of the IN-i, IN-ii, IN-iii and IN-iv surfaces so as to insight into the wettability conversion in air. CasaXPS software (Version 2.3.19, Casa Software Ltd.) was used to analyze the received data. 

## 3. Results and Discussion

### 3.1. Surface Morphology

**Figure 2:**
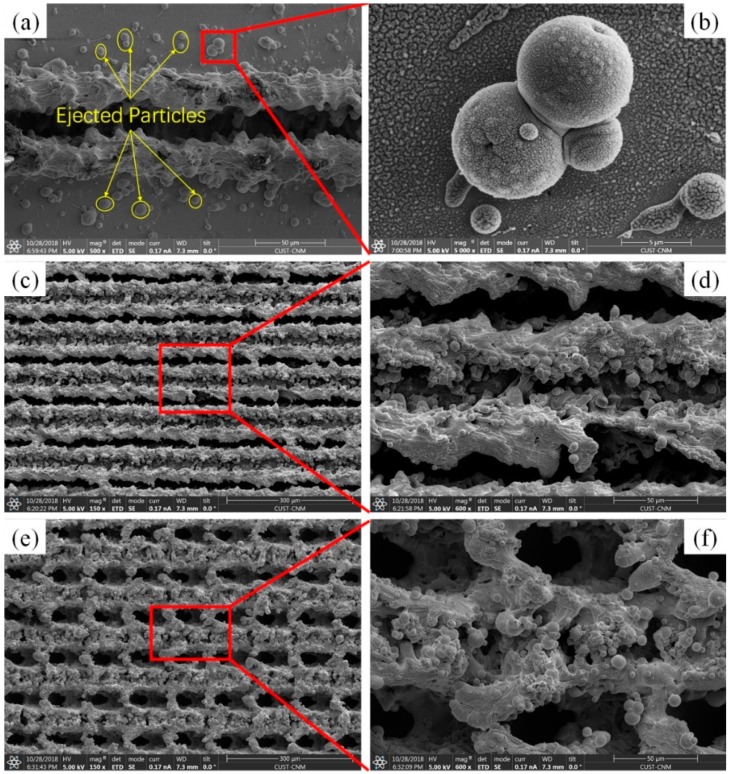
SEM images: (**a**) single groove ablated by nanosecond laser, (**c**) line-patterned surface, (**e**) grid-patterned surface. (**b**,**d**,**f**) Corresponding high-magnified SEM images.

The dashed lines in Figure 1 illustrates the movement of laser pulse, and the pulse distance can be calculated by the equation: *d* = *V*/*f* with *f* as the repetition rate and *V* as the scanning speed [44]. The laser pulse number of 24 per spot can be obtained by the used laser parameters shown in Table 2. In order to investigate the laser ablation process, we just fabricated a single groove on the IN718 surface as shown in Figure 2a. The typical SEM images show a distinct μ-channel was formed by the ablation of confined region, which resulted in the localized melting, evaporation and solidification in sequence. Meanwhile, it can be seen from Figure 2b that many irregular particles with the size from several hundred nanometer to micrometer were created and redeposited on the brim of the formed channel or the pristine substrate. We conjecture that the formation of this nano/micro particles was due to the ejection effect during the laser ablation process [45,46].

Figure 2c,e represent the SEM images of the line- and grid-patterned surfaces after laser ablation, and their corresponding high-magnified image are shown in Figure 2d,f, respectively. It is noted that many micro-scaled μ-channels with nano-scaled irregular particles were formed, creating the hierarchical micro/nano structures on the line-patterned IN718 substrate. Obviously, due to the overlap of bidirectional laser scanning, the grid-patterned surface experienced more serious ablation effect, causing all the ablated regions were covered by re-solidified materials. Compared with the line-patterned surface, no virgin IN718 area was observed on the grid-patterned surface. The high magnified SEM image also shows large numbers of submicron or nano particles covered on the laser-induced pillars. The generation of hierarchical topographies was due to laser ablation which can cause an increase temperature on the IN718 interface because of the absorption of laser energy. The μ-channels or μ-pillars combined with many irregular particles generated the hierarchical surface structures, leading to the increase of average surface roughness. As a result, many air pockets can be trapped underneath the water droplet, decreasing the contact area between liquid droplet and the laser-induced surfaces.

**Figure 3:**
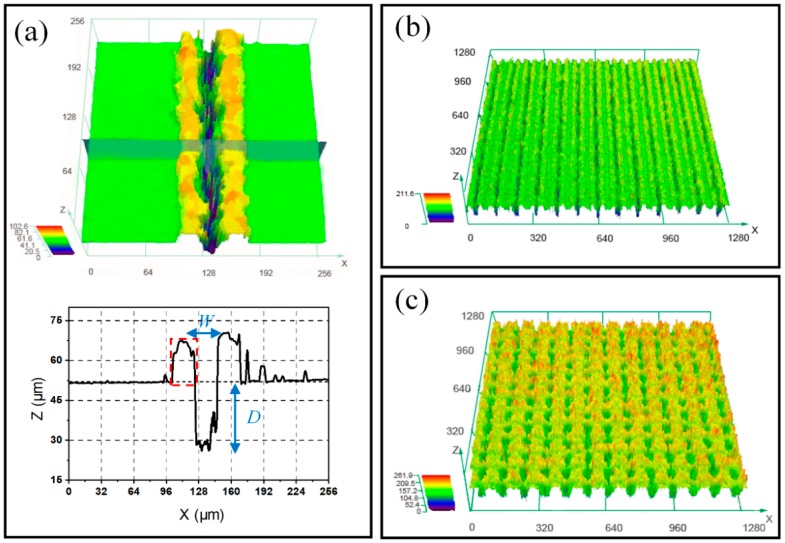
(**a**) 3D and cross-sectional profile of laser ablated single groove, 3D profile of the as-prepared surface: (**b**) line pattern, (**c**) grid-pattern.

3D profiles and average surface roughness (*R*_a_) of the laser ablated samples with line and grid patterns were further measured using laser confocal microscopy. Figure 3a demonstrates that the average width (*W*) of the laser ablated single μ-groove was 29 ± 5.8 μm and the average depth (*D*) was 25 ± 3.2 μm. It is clear from cross-sectional image that after laser ablation process, the molten materials were resolidified on the brims of the ablated groove (as shown the red box in Figure 3a). Therefore, the surface morphology changed significantly after laser ablation process, and as-prepared surfaces became obviously rough. 3D profiles of laser-induced surfaces with line and grid pattern are displayed in the Figure 3b,c, respectively. It is noted that the grid-patterned surface was much rougher than the line-patterned one, which can be proved by the average surface roughness values (*R*_a_). The results indicate that the as-prepared surface with line pattern had an average surface roughness value of 18 ± 1.2 μm, and such roughness value on grid-patterned surface was increased to 26 ± 1.2 μm. According to the Wenzel theory, the surface roughness has a close relationship with the apparent contact angle. In the following section, the initial super-hydrophilicity will be extensively discussed in terms of the surface roughness.

### 3.2. Wettability

**Figure 4:**
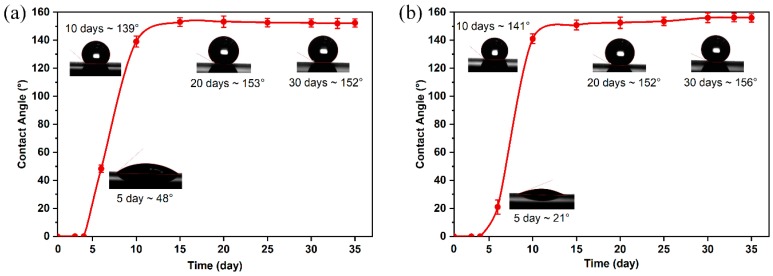
Variations of apparent contact angles (APCAs) for the as-fabricated surfaces with (**a**) line-patterned structure and (**b**) grid-patterned structure.

The line- and grid-patterned surfaces were exposed to ambient air after laser ablation, and surface wetting property was evaluated through measuring the APCAs. The increase of APCA implies the increment of hydrophobicity. The time-dependent APCAs for the air-exposed patterned surfaces are shown in Figure 4. The fresh pristine IN718 surface (IN-i) presented hydrophilic property since its APCA was only 45.2 ± 0.6°. The aged pristine IN718 surface (IN-ii) also showed a hydrophilic character with the APCA of 49.5 ± 3.5°, almost the same with the fresh polished surface (IN-i), indicating that the surface wettability was not obviously changed even though the polished IN718 surface had been stored in the ambient air for long period. However, the initial as-fabricated IN718 surfaces exhibited super-hydrophilic character immediately after laser ablation. When the liquid contacted the laser-induced hierarchical rough surface, the water droplet could quickly spread out and was almost completely penetrated into the surface channels. It was therefore unable to obtain APCA value. Within 4 days after laser ablation, this super-hydrophilic state on fresh laser-induced IN718 surfaces was particularly pronounced. The initial conversion from original hydrophilic to super-hydrophilic was possibly due to the modification of surface morphology. The amplified hydrophilicity can be described by Wenzel theory in terms of increase of average surface roughness value [47].

(1) $$\cos\theta_{w}=\mathrm{rcos}\theta_{0}$$

where *θ*_w_ denotes the APCA of the laser-induced rough surface. Because of the difficulties to prepare the ideal solid surface, *θ*_0_ is in accordance to the APCA of the fresh polished IN718 surface (i.e., the smooth IN-i surface). *r* > 1 is the average surface roughness value (*R*_a_). Equation (1) implies that with the increment of surface roughness, the APCA will decline for a pristine hydrophilic surface. Conversely, the APCA will grow for a pristine hydrophobic surface. In terms of hydrophilic IN718 material, at early stage of wettability evolution, the fresh line- and gird-patterned surface showed ultralow APCA due to the enhancement of average surface roughness. In addition, the oxidation effect during the laser ablation process could result in a layer of surface oxide. This may be another possible reason to explain the occurrence of initial super-hydrophilicity because the produced oxide has a hydrophilic nature, which will be discussed later.

It is noted that after 5 days air exposure, APCAs of the two patterned surfaces started to grow. Interestingly, on the Day 5, the line-patterned surface showed a larger APCA comparing with the grid-patterned surface, which resulted from the difference of surface topography. This is because the grid-patterned surface presented no virgin IN718 materials left, leading to a higher surface polarity due to more serious laser ablation effect. On the contrary, some virgin areas presented on the line-patterned surface can slightly balance the surface polarity after the laser ablation. From Day 5 to Day 10, the APCAs witnessed a sharp increase to around 140°. Posteriorly to 15 days, the APCA of line- and grid-patterned surfaces showed slight increase. Over a period of 20 days, the growing trend stopped. The as-prepared surfaces showed relatively steady APCAs in the following days. Over 30 days air exposure, the ablated surfaces showed typical super-hydrophobicity with the relative steady APCA of 152.3 ± 1.2° for line-patterned surface, and 156.8 ± 1.1° for the grid-patterned surface. In addition, the line-patterned surface showed a contact angle hysteresis of 7.6 ± 1.0° (advancing contact angle: 155.2 ± 1.0° and receding contact angle: 147.6 ± 1.0°). The grid-patterned super-hydrophobic surface had a relative smaller contact angle hysteresis of 6.2 ± 1.0° (advancing contact angle: 159.6 ± 1.0° and receding contact angle: 153.4 ± 1.0°). It is revealed that the aged laser-induced samples presented the unique super-hydrophobic property due to the high APCA greater than 150° and low contact angle hysteresis less than 10° [48,49]. Therefore, it is concluded that after laser treatment, the as-prepared surfaces presented initial super-hydrophilicity, then slowly reached super-hydrophobic state in ambient air over 30 days. The wettability conversion mechanism and the difference of stable APCAs between the line- and grid-patterned surfaces will be extensively discussed in Section 3.4.

### 3.3. Surface Chemistry

During the laser ablation process, surface oxidation reaction was inevitable, resulting in the formation of oxide layer on the laser treated samples. Previous literatures have clearly demonstrated that the formation of hydrophilic oxide layer will produce large number of surface defects. Therefore, the created oxide layer is not wanted because it will considerably increase surface free energy and prolong the interval for the laser-induced surfaces to reach super-hydrophobic property [44]. In this study, we mainly focus on the insights into the slow wettability conversion on the line- and grid-patterned surfaces after being exposed to the ambient air.

**Figure 5:**
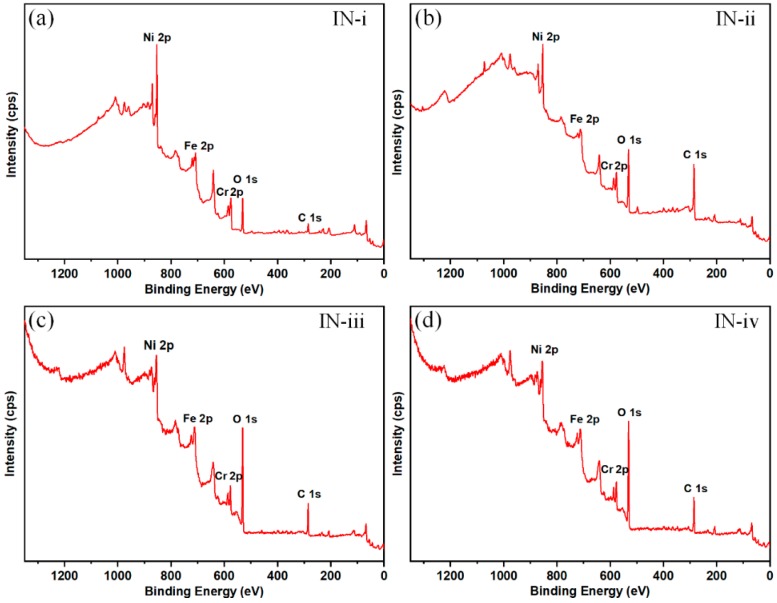
XPS spectra of survey scan: (**a**) fresh pristine IN718 surface, (**b**) aged pristine IN718 surface (**c**) line-patterned super-hydrophobic surface, (**d**) grid-patterned super-hydrophobic surface [23]. Figure 5c,d are reproduced with permission from Elsevier.

It has been reported that growth of surface carbon, which can reduce surface polarity, plays an important role in the air-exposed wettability conversion to super-hydrophobicity [36,44]. Therefore, the mechanism of time-dependent wettability was investigated though analyzing surface chemical compositions of pristine IN-i and IN-ii surfaces, super-hydrophobic IN-iii and IN-iv surfaces using XPS. The XPS spectra and the corresponding surface elemental components are shown in Figure 5 and Table 3, respectively. The displayed spectra indicate that three main metallic peak signals of Ni 2p, Cr 2p and Fe 2p were presented on these surfaces. Besides, C 1s and O 1s peaks appeared at approximately 285.0 eV and 531.5 eV, respectively.

It is noted from Table 3 that the fresh pristine surface (IN-i) possessed over 20% carbon, instead of zero, indicating that the fresh surface was not free of carbon and was deposited by some contaminants containing carbon element. We conjecture that the deposited carbon possibly came from two key sources: the absorbed organic matters before XPS measurement and the residual oil in the XPS vacuum chamber. Compared to the fresh pristine surface (IN-i), the relative carbon content on the as-prepared super-hydrophobic surfaces (IN-iii and IN-iv) showed significant increase after the patterned surfaces were exposed to air over 30 days. Correspondingly, the relative oxygen and metallic elements declined since the total content of carbon, oxygen and metallic elements was 100%. Stunningly, the carbon content on the aged pristine IN718 surface (IN-ii) considerably rose to 61.29% when exposed to ambient air for over one year, which implies that a vast of complex contaminants having large amount of carbon were absorbed on the flat IN718 surface. It is believed that the surface morphology showed no obvious change during the exposure to air condition. Thus, the surface wettability conversion was related with the modification of surface chemistry. The growth of surface carbon content was due to the attachment of airborne hydrocarbon contaminants from air moisture, causing the reduction of the surface free energy. It can be concluded that the surface hydrophobicity was enhanced due to the combination of surface hierarchical structures and the reduction of surface free energy. Therefore, the relative content of absorbed airborne hydrocarbon can be characterized by the amount of surface carbon. The deconvolution of C 1s was performed utilizing the software of CasaXPS in order to further analyze the carbon state and explore the chemisorption mechanism of airborne hydrocarbon.

**Figure 6:**
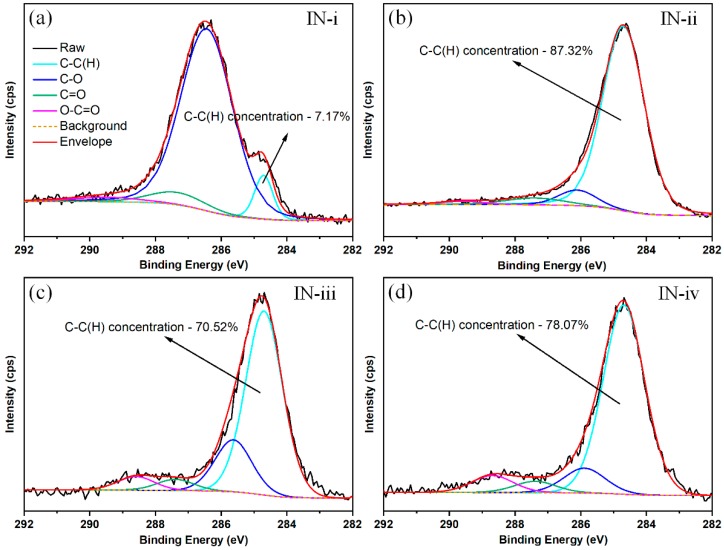
Deconvolutions of C 1s peaks and their corresponding relative concentration of C–C(H) group on the investigated surfaces: (**a**) fresh pristine IN718 surface, (**b**) aged pristine IN718 surface (**c**) line-patterned super-hydrophobic surface, (**d**) grid-patterned super-hydrophobic surface.

Here, the deconvolution of C 1s spectra presented four contributions as shown in Figure 6. One contribution located at around 284.7 eV was assigned to hydrocarbon chains or graphitic structure (C–C(H)). The C–O contribution was related with the alcohols/ether, the C=O functional group corresponded to aldehydes/ketones, and the O–C=O bond contributed to carboxyl/ester matters. The binding energies at approximately 289.6, 287.5, and 285.6 eV were corresponding to the functional group of O–C=O, C=O and C–O, respectively. Generally, C–C(H) is considered as the nonpolar bond, contributing to the increase of surface hydrophobicity. Otherwise, ether, carbonyl and carboxyl present hydrophilic property because of high polarity [50]. Therefore, the relative amount of C–C(H) bond can be used as an indicator to characterize the surface wetting property.

In addition, the relative concentrations of C–C(H) bond on the studied surfaces were compared. The results indicate that the fresh pristine surface (IN-i) presented very small amount of C–C(H) content and thus polar sites were the dominance of surface free energy, resulting in the hydrophilic property. However, although the aged flat surface (IN-ii) showed hydrophilic property, it possessed the highest C–C(H) concentration of 87.32% among the four samples. The accumulation of organic contaminants contributed to the large amount of C–C(H) concentration on the aged pristine substrate. When the line- and grid-patterned samples with unique surface texture were stored in ambient air over 30 days, their relative C–C(H) concentrations were both over 70%, which could contribute to the super-hydrophobicity due to the dominance of the nonpolar sites. Interestingly, slight difference of C–C(H) concentration is noticed between the line-patterned surface (70.52%) and grid-pattered surface (78.07%). We conjecture that the higher C–C(H) concentration could account for the larger APCA on the as-prepared super-hydrophobic surfaces.

It is reported that there are various organic matters possessing nonpolar short chain molecules, for example, acetic acid, formic and methanol [51]. Thanks to the carboxylation effect, surface free energy can be continuously decreased due to the gradual chemisorption of nonpolar chains from air moisture. The time-dependent wettability conversion on the as-prepared surfaces can be ascribed to this depolarization effect. This aspect will be extensively discussed in the following section.

### 3.4. Wettability Conversion Mechanism

The formation of a carbonaceous film with nonpolar sites will significantly reduce surface polarity, making the laser-induced surfaces more hydrophobic. The attached airborne contaminants originating from air moisture, such as acetic acids, formic and polymeric hydrocarbons, could introduce short nonpolar molecules and thus decrease surface free energy. In this process, surface hydroxylation is regarded to play a key role in the chemisorption mechanism. Laser ablation can produce many unsaturated metal and oxygen atoms which served as strong Lewis acid and base pairs, respectively [52]. Once reacting with the dissociated OH^−^ and H^+^ from interfacial water vapor molecules, the Lewis acid and base pairs will be quickly hydroxylated, leading to the reduction of surface free energy due to the weakness of Lewis acid-base sites. However, surface hydroxylation cannot elucidate the slow wettability conversion as the hydroxylation reaction is very fast. On the other hand, the surface with hydroxides present highly adhesive property to water molecules, implying that the hydroxylation effect is not the reasonable explanation to elucidate the gradual wettability conversion.

However, the hydroxyls generated in the hydroxylation process can absorb the carboxylates (for instance, acetic acids, formic) and other airborne organic matters (such as polymeric hydrocarbons). Once these organic matters reacted with hydroxyls, their nonpolar chain *R* containing C–C(H) functional groups can be chemisorbed onto the laser-induced surfaces, leading to the reduction of surface polarity [53]. The corresponding chemical reaction can be described by the following Equation (2):

(2) $$\mathrm{Ni}-\mathrm{OH}+\mathrm{RCOOH}(g)\to {\mathrm{RCOO}-\mathrm{Ni}+H}_{2}O$$

This chemisorption process to depolarize surface energy is relatively slow and therefore can elucidate the step-by-step wettability conversion from initial super-hydrophi licity to final super-hydrophobicity when the laser-induced surfaces were stored in ambient air. The concentration of O–C=O group can further verify this assumption. The results infer that on the IN-i surface, the O–C=O concentration was only 2.39%. However, 30 days later, the relative concentration of O–C=O bond dramatically rose to 5.24% for the line-patterned surface, and to 7.01% for the grid-patterned surface. Although the polar O–C=O functional group was also attached on the laser ablated surfaces, these surfaces were dominated by the nonpolar C–C(H) groups. It is therefore possible for the laser patterned surfaces to show super-hydrophobicity. The results confirm that the condensation reaction is conducive to the gradual super-hydrophobicity due to the absorption of nonpolar molecules, which leads to the slow reduction of surface polarity. Remarkably, although the aged pristine IN718 sample with flat surface structure had the largest carbon content as well as the highest relative C–C(H) concentration, it still presented the hydrophilic property. Thus, except for the modification of surface chemistry, the laser-induced wettability conversion should be explored further based on surface topography.

Previous literatures proved that the coating of low-free-energy substance on flat surfaces cannot ensure the super-hydrophobicity [54,55], implying that surface chemical modification is not the only factor to determine surface super-hydrophobicity. Obviously, the difference of the aged pristine sample (IN-ii) and the as-prepared super-hydrophobic surfaces (IN-iii and IN-iv) is the surface roughness. Therefore, the influence of surface roughness on the wettability conversion mechanism should be investigated in detail.

**Figure 7:**
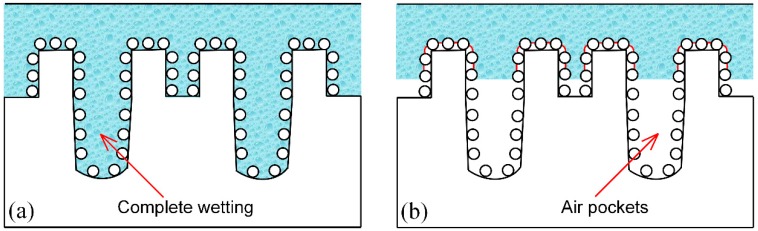
The wetting model of the water droplet on the laser-induced surfaces with hierarchical topographies: (**a**) initial super-hydrophilic surface, (**b**) aged super-hydrophobic surface.

In terms of the initial super-hydrophilicity, surface roughness of the laser-induced samples significantly increased due to the formation of μ-channels with micro/nano particles. In this case, the laser ablated surfaces can be modeled by Wenzel state (as shown in Figure 7a), inferring that with the increase of surface roughness, the contact angle will decline regarding the pristine hydrophilic IN718 surface. Thus, the created hierarchical topographies amplified the effect of surface hydrophilicity, leading to super-hydrophilicity immediately after laser ablation. After stored in air for over 30 days, the line- and gird-patterned surfaces reached super-hydrophobic state due to the chemisorbed nonpolar organic molecules. Many studies utilized following simplified Cassie-Baxter equation to explain the conversion of super-hydrophobicity [56,57,58,59]:

(3) $${\cos\theta }_{c}=f_{s}(1+{\cos\theta }_{0})-1$$

where *θ*_0_ presents the APCA of the polished surface, and *θ*_c_ is the APCA of the laser-induced rough surface. *f_s_* denotes the portion of solid/liquid area. However, this oversimplified Cassie-Baxter Equation (3) is not suitable to elucidate the wetting state of the aged super-hydrophobic surfaces due to the presence of laser-induced hierarchical topographies. According to the SEM and 3D images of the laser-induced surface, more accurate wetting state can be shown in Figure 7b. Based on previous papers, it has been both experimentally and theoretically proved that the hierarchical multi-scale topography can enhance water repellency of surfaces [60,61]. This is because many air pockets were generated underneath the water droplet and the air cushion could prevent the liquid from further penetrating into the grooves. In this current study, the grid-patterned surface experienced much more serious laser ablation effect. Therefore, more air would be trapped underneath the water droplet and less solid area was wetted by water, which resulted in a better super-hydrophobicity on the grid-patterned surface. Because of the formed solid-air composite interface with low-surface-energy molecules, the dispensed water droplet could hardly penetrate into the micro/nano structures on the aged super-hydrophobic surfaces.

## 4. Conclusions

In this study, the super-hydrophobic surfaces with line or grid pattern were successfully finished on IN718 material via nanosecond laser ablation. The as-prepared surfaces initially showed super-hydrophilicity. During the air exposing period, the laser fabricated surfaces experienced gradual wettability conversion to steady super-hydrophobicity. The wettability conversion mechanism was extensively explored based on the analyses of surface morphology and surface chemistry. It can be concluded that after laser treatment, the gradual conversion of APCA related with the chemisorbed airborne contaminants from air moisture. The attached organic matters can introduce nonpolar C–C(H) bond onto the laser-induced surfaces, which can slowly lower surface free energy. In terms of surface morphology and surface roughness, the initial super-hydrophilicity was analyzed based on the Wenzel theory, and the comparison of super-hydrophobicity between the line- and grid-patterned surfaces was also elucidated. Noticeably, this study confirmed that the surface chemistry is by no means the only factor to determine surface super-hydrophobicity. The laser-induced super-hydrophobicity was attributed to the synergistic effect of the surface morphology and the surface chemistry. This research not only benefits academic to better understand the generation of air-exposed super-hydrophobic surfaces by laser, but also further inspire factories to alter surface chemistry and effectively produce durable super-hydrophobic surfaces for engineering applications.

## Figures and Tables

**Table 1:** The chemical components of IN718 alloy.

**Element**	**Ni**	**Cr**	**Nb**	**Al**	**Mo**	**Si**	**C**	**Mn**
wt. (%)	53.16	18.03	5.48	0.66	3.11	0.16	0.07	0.08
**Element**	**P**	**Co**	**Ti**	**Cu**	**B**	**Ta**	**S**	**Fe**
wt. (%)	0.015	0.23	1.15	0.07	0.0028	0.008	0.002	Balanced

**Table 2:** Laser parameters performed to ablate the IN718 substrates.

Laser Parameters	Values
Wavelength	1064 nm
Average laser power	10 W
Repetition rate	20 kHz
Pulse duration	50 ns
Laser fluence	17.69 J/cm^2^
Spacing	100 μm
Scanning speed	50 mm/s

**Table 3:** Elemental components detected by XPS.

Sample No.	C (atomic %)	O (atomic %)	Ni (atomic %)	Fe (atomic %)	Cr (atomic %)
IN-i	26.62	25.04	16.59	14.55	17.21
IN-ii	61.29	20.93	6.21	6.30	6.30
IN-iii	34.23	44.65	9.30	5.28	6.54
IN-iv	40.50	43.42	8.39	3.39	4.30

## References

[1] Venkatesan K., Ramanujam K., Kuppan P. (2016). Parametric modeling and optimization of laser scanning parameters during laser assisted machining of Inconel 718. Opt. Laser Technol..

[2] Keshavarzkermani A., Sadowski M., Ladani M. (2018). Direct metal laser melting of Inconel 718: Process impact on grain formation and orientation. J. Alloy Compd..

[3] Wang Z., Guan K., Gao M., Li X., Chen X., Zeng X. (2018). The microstructure and mechanical properties of deposited-IN718 by selective laser melting. J. Alloy Compd..

[4] Xiao L.J., Zeng W.G., Liao G.F., Yi C.F., Xu Z.S. (2018). Thermally and chemically stable candle soot superhydrophobic surface with excellent self-cleaning properties in air and oil. ACS Appl. Nano Mater..

[5] Xiao L.J., Deng M., Zeng W.G., Zhang B.X., Xu Z.S., Yi C.F., Liao G.F. (2017). Novel robust super-hydrophobic coating with self-cleaning properties in air and oil based on rare earth metal oxide. Ind. Eng. Chem. Res..

[6] Sun K., Yang H., Xue W., He A., Zhu D.H., Liu W.W., Adeyemi K., Cao Y. (2018). Anti-biofouling superhydrophobic surface fabricated by picosecond laser texturing of stainless steel. Appl. Surf. Sci..

[7] Yang Z., Liu X.P., Tian Y.L. (2019). Fabrication of super-hydrophobic nickel film on copper substrate with improved corrosion inhibition by electrodeposition process. Colloids Surf. A.

[8] Lu M.C., Lin C.C., Lo C.W., Huang C.W., Wang C.C. (2017). Superhydrophobic Si nanowires for enhanced condensation heat transfer. Int. J. Heat Mass Trans..

[9] Bhushan B., Jung Y.C. (2011). Natural and biomimetic artificial surfaces for superhydrophobicity, self-cleaning, low adhesion, and drag reduction. Prog. Mater. Sci..

[10] Wang C.Z., Tang F., Hao P.F., Li Q., Wang X.H. (2017). Experimental study on the drag reduction effect of a rotating superhydrophobic surface in micro gap flow field. Microsyst. Technol..

[11] Tian X.L., Verho T., Ras R.H.A. (2016). Moving superhydrophobic surfaces towards real-world applications. Science.

[12] Toosi S.F., Moradi S., Ebrahimi M., Hatzikiriakos S.G. (2016). Microfabrication of polymeric surfaces with extreme wettability using hot embossing. Appl. Surf. Sci..

[13] Latthe S.S., Terashima C., Nakata K., Sakai M., Fujishima A. (2014). Development of sol–gel processed semi-transparent and self-cleaning superhydrophobic coatings. J. Mater. Chem. A.

[14] He Y., Jiang C.Y., Yin H.X., Yuan W.Z. (2011). Tailoring the wettability of patterned silicon surfaces with dual-scale pillars: From hydrophilicity to superhydrophobicity. Appl. Surf. Sci..

[15] Yang X.M., Zhong Z.W., Diallo E.M., Wang Z.H., Yue W.S. (2014). Silicon wafer wettability and aging behaviors: Impact on gold thin-film morphology. Mater. Sci. Semicond. Process..

[16] Kwon M.H., Jee W.Y., Chu C.N. (2015). Fabrication of hydrophobic surfaces using copper electrodeposition and oxidation. Microsyst. Technol..

[17] Ye Y.W., Liu Z.Y., Liu W., Zhang D.W., Zhao H.C., Wang L.P., Li X.G. (2018). Superhydrophobic oligoaniline-containing electroactive silica coating as pre-process coating for corrosion protection of carbon steel. Chem. Eng. J..

[18] Crick C.R., Bear J.C., Southern P., Parkin I.P. (2013). A general method for the incorporation of nanoparticlesinto superhydrophobic films by aerosol assisted chemical vapour deposition. J. Mater. Chem. A.

[19] Zhai Z.Y., Wang W.J., Mei X.S., Li M., Cui J.L., Wang F.C., Pan A.F. (2018). Effect of the surface microstructure ablated by femtosecond laser on the bonding strength of EBCs for SiC/SiC composites. Opt. Commun..

[20] Baron C.F., Mimidis A., Puerto D., Skoulas E., Stratakis E., Solis J., Siegel J. (2018). Biomimetic surface structures in steel fabricated with femtosecond laser pulses: Influence of laser rescanning on morphology and wettability. Beilstein J. Nanotechnol..

[21] Rung S., Schwarz S., Götzendorfer B., Esen C., Hellmann R. (2018). Time dependence of wetting behavior upon applying hierarchic nano-micro periodic surface structures on brass using ultra short laser pulses. Appl. Sci..

[22] Trdan U., Hočevar M., Gregorčič P. (2017). Transition from superhydrophilic to superhydrophobic state of laser textured stainless steel surface and its effect on corrosion resistance. Corros. Sci..

[23] Yang Z., Tian Y.L., Yang C.J., Wang F.J., Liu X.P. (2017). Modification of wetting property of Inconel 718 surface by nanosecond laser texturing. Appl. Surf. Sci..

[24] Tian Y.L., Zhao Y.C., Yang C.J., Wang F.J., Liu X.P., Jing X.B. (2018). Fabrication of bio-inspired nitinol alloy surface with tunable anisotropic wetting and high adhesive ability. J. Colloid Interface Sci..

[25] Athanasiou C.E., Bellouard Y. (2015). A monolithic micro-tensile tester for investigating silicon dioxide polymorph micromechanics, fabricated and operated using a femtosecond laser. Micromachines.

[26] Dadashi S., Dadashi R., Delavari H. (2018). Optical and structural properties of Bi-based nanoparticles prepared via pulsed Nd:YAG laser ablation in organic liquids. Appl. Phys. A.

[27] Yang W.J., Gerke S.A., Ng K.W., Rao Y., Chase C., Chang-Hasnain C.J. (2015). Laser optomechanics. Sci. Rep..

[28] Wang Z.K., Zhang H.Y., Lim R.Y.H., Wang Z.F., Lam Y.C. (2011). Improving surface smoothness of laser-fabricated microchannels for microfluidic application. J. Micromech. Microeng..

[29] Wang Z.K., Zheng H.Y., Xia H.M. (2011). Femtosecond laser-induced modification of surface wettability of PMMA for fluid separation in microchannels. Microfluid. Nanofluid..

[30] Phillips K.C., Gandhi H.H., Mazur E., Sundaram S.K. (2015). Ultrafast laser processing of materials: A review. Adv. Opt. Photonics.

[31] Yan H.P., Rashid M.R., Khew S.Y., Li F.P., Hong M.H. (2018). Wettability transition of laser textured brass surfaces inside different mediums. Appl. Surf. Sci..

[32] Sun C., Zhao X.W., Han Y.H., Ze Z. (2008). Control of water droplet motion by alteration of roughness gradient on silicon wafer by laser surface treatment. Thin Solid Films.

[33] Kietzig A.M., Mirvakili M.N., Kamal S., Englezos P., Hatzikiriakos S.G. (2011). Cassie to Wenzel wetting transitions on femtosecond laser-patterned pure metallic substrates. J. Adhes Sci. Technol..

[34] Yang C.J., Mei X.S., Tian Y.L., Zhang D.W., Li Y., Liu X.P. (2016). Modification of wettability property of titanium by laser texturing. Int. J. Adv. Manuf. Technol..

[35] Rung S., Schwarz S., Zettl J., Götzendorfer B., Esen C., Hellmann R. (2018). Static and dynamic contact angle of water influenced by femtosecond laser based ripple structures on metals. J. Laser Micro Nanoen..

[36] Yang Z., Liu X.P., Tian Y.L. (2019). Insights into the wettability transition of nanosecond laser ablated surface under ambient air exposure. J. Colloid Interface Sci..

[37] Eral H.B., Oh J.M. (2013). Contact angle hysteresis: A review of fundamentals and applications. Colloid. Polym. Sci..

[38] Zeng W.G., Chen J., Yang H., Deng L.D., Liao G.F., Xu Z.S. (2017). Robust coating with superhydrophobic and self-cleaning properties in either air and oil based on natural zeolite. Surf. Coat. Technol..

[39] Long J.Y., Zhong M.L., Zhang H.J., Fan P.X. (2015). Superhydrophilicity to superhydrophobicity transition of picosecond laser microstructured aluminum in ambient air. J. Colloid Interface Sci..

[40] Rajab F.H., Liu Z., Li L. (2019). Long term superhydrophobic and hybrid superhydrophobic/superhydrophilic surfaces produced by laser surface micro/nano surface structing. Appl. Surf. Sci..

[41] Marmur A., Della V.C., Siboni S., Amirfazli A., Drelich J.W. (2017). Contact angles and wettability: Towards common and accurate terminology. Surf. Innov..

[42] Marmur A. (2006). Soft contact: Measurement and interpretation of contact angles. Soft Matter.

[43] Huhtamäki T., Tian X.L., Korhonen J.T., Ras R.H.A. (2018). Surface-wetting characterization using contact-angle measurements. Nat. Protoc..

[44] Cardoso J.T., Garcia-Girón A., Romano J.M., Huerta-Murillo D., Jagdheesh R., Walker M., Dimov S.S., Ocaña J.L. (2017). Influence of ambient conditions on the evolution of wettability properties of an IR-, ns-laser textured aluminium alloy. RSC Adv..

[45] Farid N., Harilal S.S., EI-Atwani O., Ding H., Hassanein A. (2014). Experimental simulation of materials degradation of plasma-facing components using lasers. Nucl. Fusion.

[46] Besozzi E., Maffini A., Dellasega D., Russo V., Facibeni A., Pazzaglia A., Beghi M.G., Passoni M. (2018). Nanosecond laser pulses for mimicking thermal effects on nanostructured tungsten-based materials. Nucl. Fusion.

[47] Wenzel R.N. (1936). Resistance of solid surfaces to wetting by water. J. Ind. Eng. Chem..

[48] Bormashenko E. (2013). Wetting of Real Surfaces.

[49] Fadeeva E., Truong V.K., Stiesch M., Chichkov B.N., Crawford R.J., Wang J., Ivanova E.P. (2011). Bacterial retention on superhydrophobic titanium surfaces fabricated by femtosecond laser ablation. Langmuir.

[50] Long J.Y., Zhong M.L., Fan P.X., Gong D.W., Zhang H.J. (2015). Wettability conversion of ultrafast laser structured copper surface. J. Laser Appl..

[51] Dabek-Zlotorzynska E., Celo V. (2008). Capillary Electrophoresis.

[52] Li Z.T., Wang Y.J., Kozbial A., Shenoy G., Zhou F., McGinley R., Ireland P., Morganstein B., Kunkel A., Surwade S.P. (2013). Effect of airborne contaminants on the wettability of supported graphene and graphite. Nat. Mater..

[53] Drzymala J. (1994). Hydrophobicity and collectorless flotation of inorganic materials. Adv. Colloid Interface.

[54] Su F.H., Yao K. (2014). Facile fabrication of superhydrophobic surface with excellent mechanical abrasion and corrosion resistance on copper substrate by a novel method. ACS Appl. Mater. Interfaces.

[55] Bryuzgin E.V., Klimov V.V., Repin S.A., Navrotskiy A.V., Novakov I.A. (2017). Aluminum surface modification with fluoroalkyl methacrylate-based copolymers to attain superhydrophobic properties. Appl. Surf. Sci..

[56] Baldacchini T., Carey J.E., Zhou M., Mazur E. (2006). Superhydrophobic surfaces prepared by microstructuring of silicon using a femtosecond laser. Langmuir.

[57] Boinovich L.B., Emelyanenko A.M., Pashinin A.S., Lee C.H., Drelich J., Yap Y.K. (2012). Origins of Thermodynamically stable superhydrophobicity of Boron Nitride nanotubes coatings. Langmuir.

[58] Muller F.A., Kunz C., Graf S. (2016). Bio-inspired functional surfaces based on laser-induced periodic surface structures. Materials.

[59] Herminghaus S. (2000). Roughness-induced non-wetting. Europhys. Lett..

[60] Bormashenko E., Stein T., Whyman G., Bormashenko Y., Pogreb R. (2006). Wetting properties of the multiscaled nanostructured polymer and metallic superhydrophobic surfaces. Langmuir.

[61] Starostin A., Valtsifer V., Strelnikov V., Bormashenko E., Grynuov R., Bormashenko Y., Gladkikh A. (2014). Robust technique allowing the manufacture of superoleophobic (omniphobic) metallic surfaces. Adv. Eng. Mater..

